# Metastasis to para-aortic lymph nodes cephalad to the renal veins in patients with ovarian cancer

**DOI:** 10.1186/s12957-020-01841-8

**Published:** 2020-04-01

**Authors:** Shinichi Komiyama, Masaru Nagashima, Tomoko Taniguchi, Takayuki Rikitake, Mineto Morita

**Affiliations:** 1 Department of Obstetrics and Gynecology, Toho University Faculty of Medicine, 6-11-1, Omori-nishi, Ota-ku, Tokyo, 143-8541 Japan; 2 Department of Gynecology, Toho University Ohashi Medical Center, Tokyo, Japan

**Keywords:** Epithelial ovarian cancer, Supra-renal para-aortic node metastasis, Extended PAN dissection, FIGO stage

## Abstract

**Background:**

In patients with epithelial ovarian cancer, whether metastasis to para-aortic lymph nodes located cephalad to the renal veins (supra-renal PAN) should be classified as regional lymph node metastasis or distant metastasis remains controversial. This study was a preliminary retrospective evaluation of the pattern of supra-renal PAN metastasis in patients with epithelial ovarian cancer.

**Methods:**

The subjects were 25 patients with epithelial ovarian cancer, primary peritoneal cancer, or fallopian tube cancer who underwent systematic dissection of the para-aortic nodes, including the supra-renal PAN, and pelvic lymph nodes (PLN). Patient factors, perioperative factors, the number of dissected lymph nodes, and pathological lymph node metastasis were investigated.

**Results:**

Supra-renal PAN metastasis was found in 4/25 patients (16.0%). None of the 14 patients with pT1 or pT2 disease had supra-renal PAN metastasis, while 4/11 patients (36.4%) with pT3 or ypT3 disease had such metastases. None of the patients had isolated supra-renal PAN metastasis, while patients with supra-renal PAN metastasis also had multiple metastases to the infra-renal PAN and PLN.

**Conclusions:**

In patients with epithelial ovarian cancer, supra-renal PAN metastases might be considered to be distant rather than regional metastases. Further studies are needed to better define the clinical significance of supra-renal PAN metastasis.

## Introduction

The para-aortic lymph nodes (PANs) are located around the abdominal aorta and inferior vena cava and are the regional lymph nodes of the intraperitoneal organs. These nodes can be broadly classified into supra-renal PANs located cephalad (superior) to the renal veins and infra-renal PANs located caudad (inferior) to the renal veins, while the infra-renal PANs can be further classified into nodes located between the renal veins and the inferior mesenteric artery and nodes located between the inferior mesenteric artery and the aortic bifurcation [1].

In patients with epithelial ovarian cancer, primary peritoneal cancer, or fallopian tube cancer, the clinical implications of supra-renal PAN metastasis are unclear. According to the International Federation of Gynecology and Obstetrics (FIGO) staging classification, the supra-renal PANs are classified as regional lymph nodes, but it is stated that there is controversy regarding whether supra-renal PAN metastasis should be considered as regional lymph node metastasis (stage III) or distant metastasis (stage IV), and this issue should be investigated further in the future [2]. The chief reason why the clinical significance of supra-renal PAN metastasis remains unclear in patients with epithelial ovarian cancer, including primary peritoneal cancer and fallopian tube cancer, is that metastasis to these nodes has received little attention in the past.

It is possible that supra-renal PAN metastases have not been assessed in many previous studies because these nodes lie dorsal to the pancreas and dissection is difficult due to this anatomic location. In addition, the target region for systematic PAN dissection is below the renal veins in patients with ovarian cancer and dissection of the supra-renal PANs is unusual.

However, the supra-renal PANs can be sites of metastasis or recurrence in patients with advanced ovarian cancer. To determine the optimal surgical management for patients with supra-renal PAN involvement, we have tried various approaches to supra-renal PAN dissection, and we have developed a safe method for dissection of the entire PAN region, including the supra-renal PANs (extended PAN dissection). This method involves complete mobilization of the entire small intestine (including the duodenum) and the right hemicolon to expose the inferior vena cava and abdominal aorta below the epiploic foramen on the inferior surface of the liver. Consequently, it is possible to expand the para-aortic region located cephalad to the renal artery, and it is easy to dissect the supra-renal PANs. Our method is a modification of Kocher’s maneuver for duodenal mobilization and has been named Komiyama’s maneuver for dissection of high para-aortic lymph nodes [3].

We performed the present study to evaluate the pattern of supra-renal PAN metastasis and the feasibility of performing extended PAN dissection in patients with epithelial ovarian cancer, primary peritoneal cancer, or fallopian tube cancer.

## Materials and methods

### Patients

The subjects of this study were patients with an initial diagnosis of epithelial ovarian cancer, primary peritoneal cancer, or fallopian tube cancer who underwent extended PAN dissection and pelvic lymph node dissection at Toho University Ohashi Medical Center.

The eligibility criteria for our surgical procedure were as follows: (1) preoperative Eastern Cooperative Oncology Group (ECOG) Performance Status of 0–1; (2) adequate major organ function as evaluated by hematology tests, biochemical tests, coagulation tests, chest and abdominal X-ray examination, and electrocardiography; and (3) written informed consent to surgery provided by the patient. Patients who met any of the following criteria were excluded: (1) a history of intestinal obstruction, (2) uncontrolled diabetes or hypertension, (3) deep venous thrombosis, (4) pancreatic disease, (5) a desire to preserve fertility, (6) borderline malignant tumor, and (7) active double cancer.

The chest and abdomen, including the supra-renal PANs, were examined carefully by computed tomography (CT) before surgery. Staging laparotomy was performed in patients with a clinical diagnosis of early cancer, while primary debulking surgery (PDS) was done in patients with a clinical diagnosis of advanced cancer in whom it was considered that primary surgery would achieve an optimal outcome. In addition, interval debulking surgery (IDS) was performed after 3–4 cycles of neoadjuvant chemotherapy (NAC) with taxanes, platinum, and bevacizumab in patients with clinically suspected advanced cancer in whom it was considered that primary surgery would not be optimal.

### Surgical procedure

**Fig. 1:**
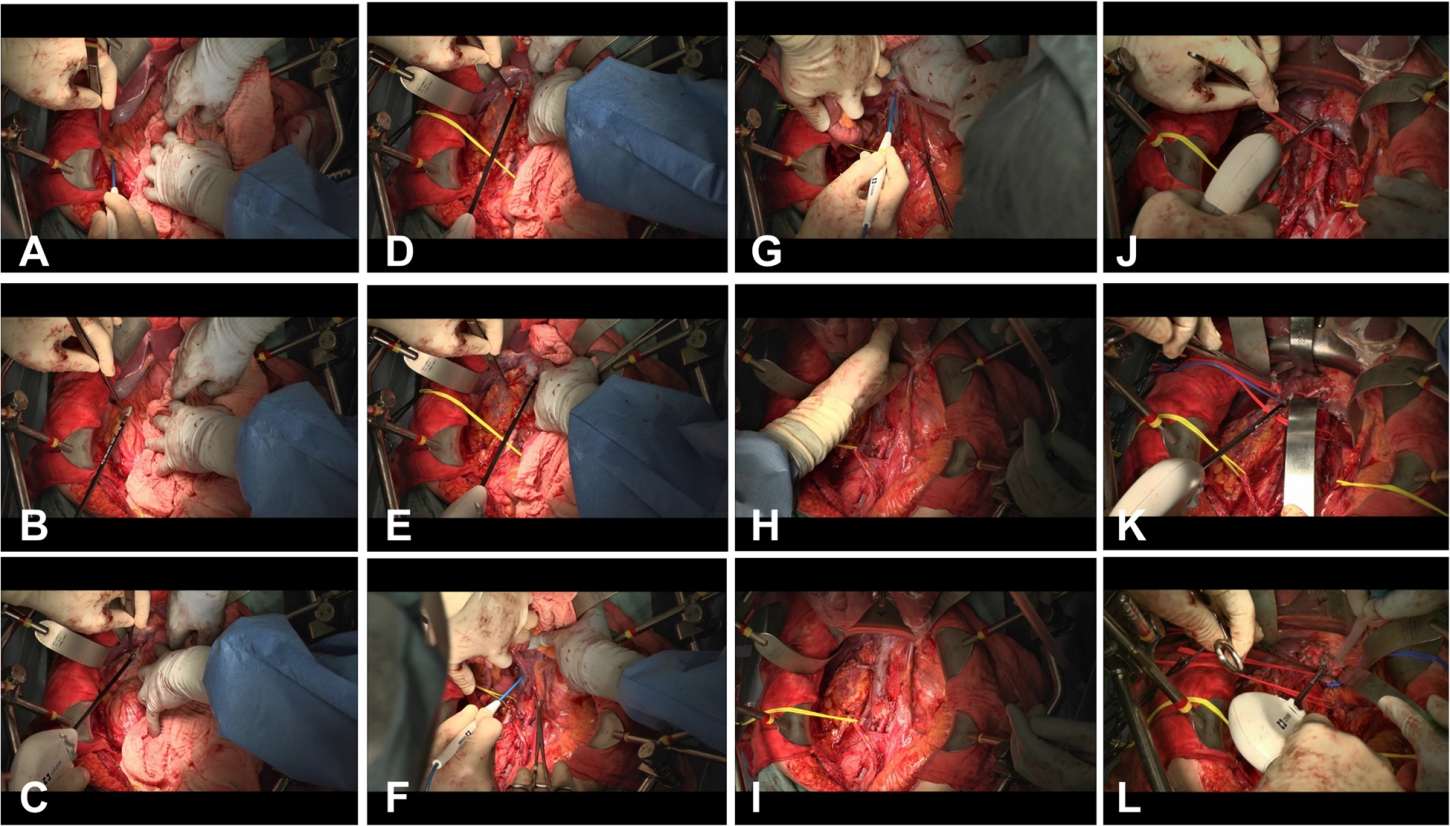
Surgical procedure of Komiyama’s maneuver for extended PAN dissection. First retroperitoneal incision was made along the so-called “Monk’s white line” (**a**, **b**). Fusion fascia was incised through the medial side of Gerota’s fascia toward the root of the right renal vein to separate the right kidney from the ascending colon, which was mobilized to expose the region from the anterior surface of the inferior vena cava to the left side of the abdominal aorta (**c**). Kocher’s maneuver was performed for mobilization of the duodenum (**d**, **e**). Second retroperitoneal incision was commenced near the bifurcation of the abdominal aorta into the common iliac arteries and was continued toward the ligament of Treitz, allowing complete mobilization of the small intestine and the right hemicolon (**f**–**h**). After mobilization, the small intestine and right hemicolon were placed in an isolation bag and lifted out of the abdominal cavity (**i**). After dissecting the infra-renal PANs, the left and right renal arteries and the left lower adrenal vein were identified, and the supra-renal PANs were dissected (**j**–**l**)

After pelvic lymph node dissection was completed, Komiyama’s maneuver for extended PAN dissection was carried out as follows (Fig. 1).

**Fig. 2:**
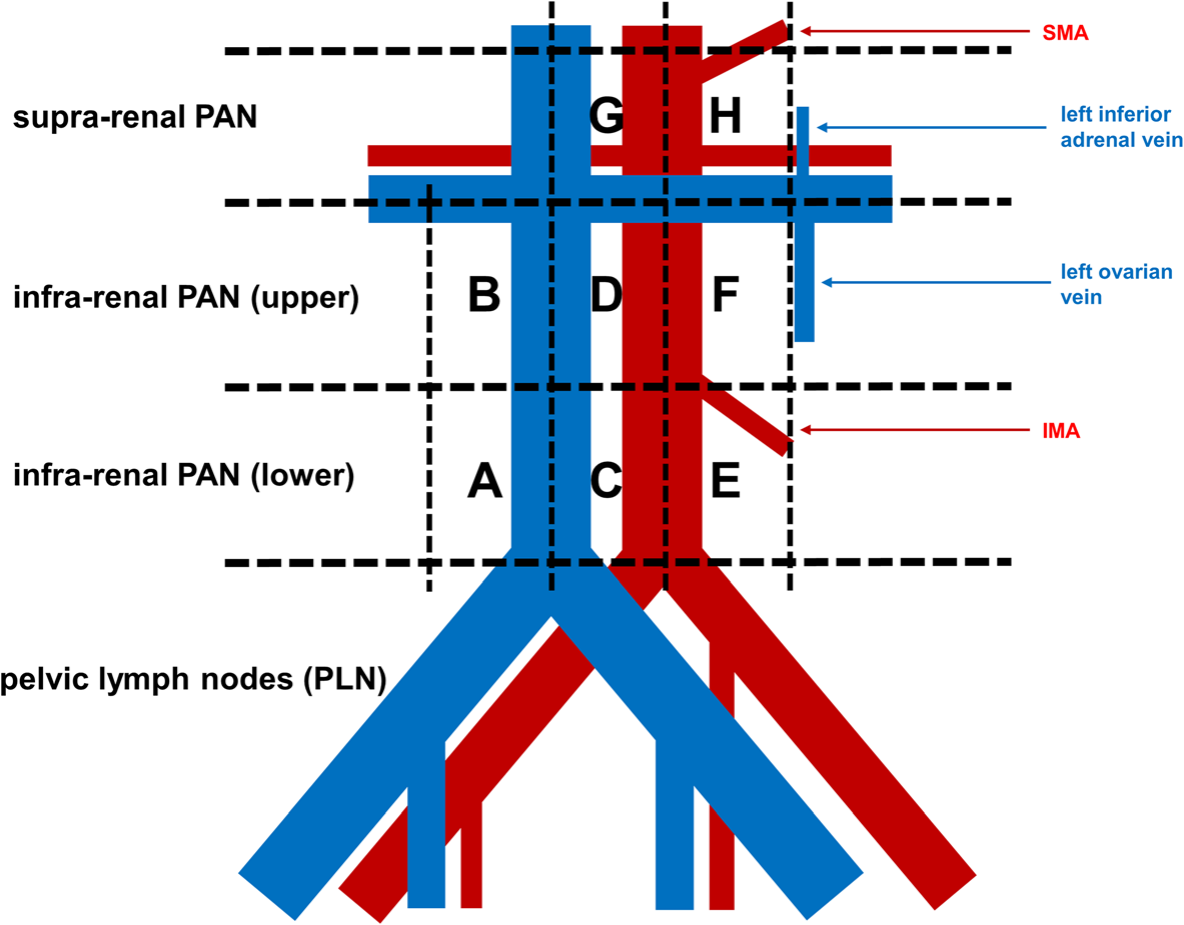
Detailed classification of the para-aortic lymph nodes. Supra-renal PANs, para-aortic lymph nodes located cephalad (superior) to the renal veins; infra-renal PANs, para-aortic lymph nodes located caudad (inferior) to the renal veins; A, infra-renal PANs located on the right side of the inferior vena cava and below the inferior mesenteric artery (IMA); B, infra-renal PANs located on the right side of the inferior vena cava and above the IMA; C, infra-renal PANs located between the inferior vena cava and the abdominal aorta and below the IMA; D, infra-renal PANs located between the inferior vena cava and the abdominal aorta and above the IMA; E, infra-renal PANs located on the left side of the aorta and below the IMA; F, infra-renal PANs located on the left side of the aorta and above the IMA; G, supra-renal PANs located between the inferior vena cava and the abdominal aorta in the region below the inferior surface of the right lobe of the liver and also below the level of the superior mesenteric artery (SMA); H, supra-renal PANs located on the left side of the aorta in the region below the level of the SMA and medial to the left adrenal gland.

(1) The ascending colon was displaced from the right paracolic gutter toward the left side. A retroperitoneal incision was made from the ileocecal region in the cephalic direction along the so-called Monk’s white line on the dorsal side of the ascending colon. At this site, Gerota’s fascia, the ascending mesocolon, and the retroperitoneum coalesce to form the so-called fusion fascia, which continues to the mesentery on the right side of the second part of the duodenum and ends superiorly at the epiploic foramen on the inferior surface of the left lobe of the liver. The fusion fascia was incised through the medial side of Gerota’s fascia toward the root of the right renal vein to separate the right kidney from the ascending colon, which was mobilized to expose the region from the anterior surface of the inferior vena cava (IVC) to the left side of the abdominal aorta. The right ovarian vein runs through the fusion fascia and then through the pelvic cavity toward the midpoint of the IVC, and the right ureter also runs through the fusion fascia, allowing both structures to be easily identified by incising the fascia. After identifying the right ovarian vein, it was transected to expose the IVC below the renal vein (Fig. 1a–c).

(2) Kocher’s maneuver was performed for mobilization of the duodenum. The second part of the duodenum was displaced toward the left side and the incision in the fusion fascia, which was at the root of the right renal vein, was extended to the epiploic foramen. Then, connective tissues were stripped from around the duodenum, and it was mobilized to completely expose the IVC below the inferior surface of the left lobe of the liver (Fig. 1d, e).

(3) A retroperitoneal incision was commenced near the bifurcation of the abdominal aorta into the common iliac arteries and was continued along the medial border of the inferior mesenteric vein toward the ligament of Treitz, allowing complete mobilization of the small intestine and the right hemicolon. After mobilization, the small intestine and right hemicolon were placed in an isolation bag and lifted out of the abdominal cavity to clearly expose the whole aortic region from the superior mesenteric artery to the common iliac artery (Fig. 1f–i).

(4) The right and left renal arteries and the left inferior adrenal vein were identified, taking care to avoid damaging these vessels. When the left high para-aortic lymph nodes were dissected, care was also taken to avoid damaging the left adrenal gland, which is embedded in fatty tissue and is difficult to distinguish from a lymph node. The high para-aortic lymph node region contains important lymphatic channels, including the lumbar lymphatic trunk, the intestinal lymphatic trunk, and the chyle cistern. These structures must be managed carefully to prevent damage that could lead to leakage of chyle and occurrence of chylous ascites. Accordingly, both a vessel sealing system and a vascular clip (double ligation) were employed to prevent such events (Fig. 1j–l). While paying attention to these points, the para-aortic lymph nodes were dissected in the following order (Fig. 2):

- Infra-renal PANs located on the right and ventral sides of the IVC (A before B).
- Infra-renal PANs located between the IVC and the abdominal aorta and dorsal to these vessels (C before D).
- Infra-renal PANs located on the left and ventral sides of the aorta (E before F).
- Supra-renal PANs located between the IVC and the abdominal aorta and ventral to these vessels (G).
- Supra-renal PANs located on the left and dorsal sides of the aorta (H).

**Fig. 3:**
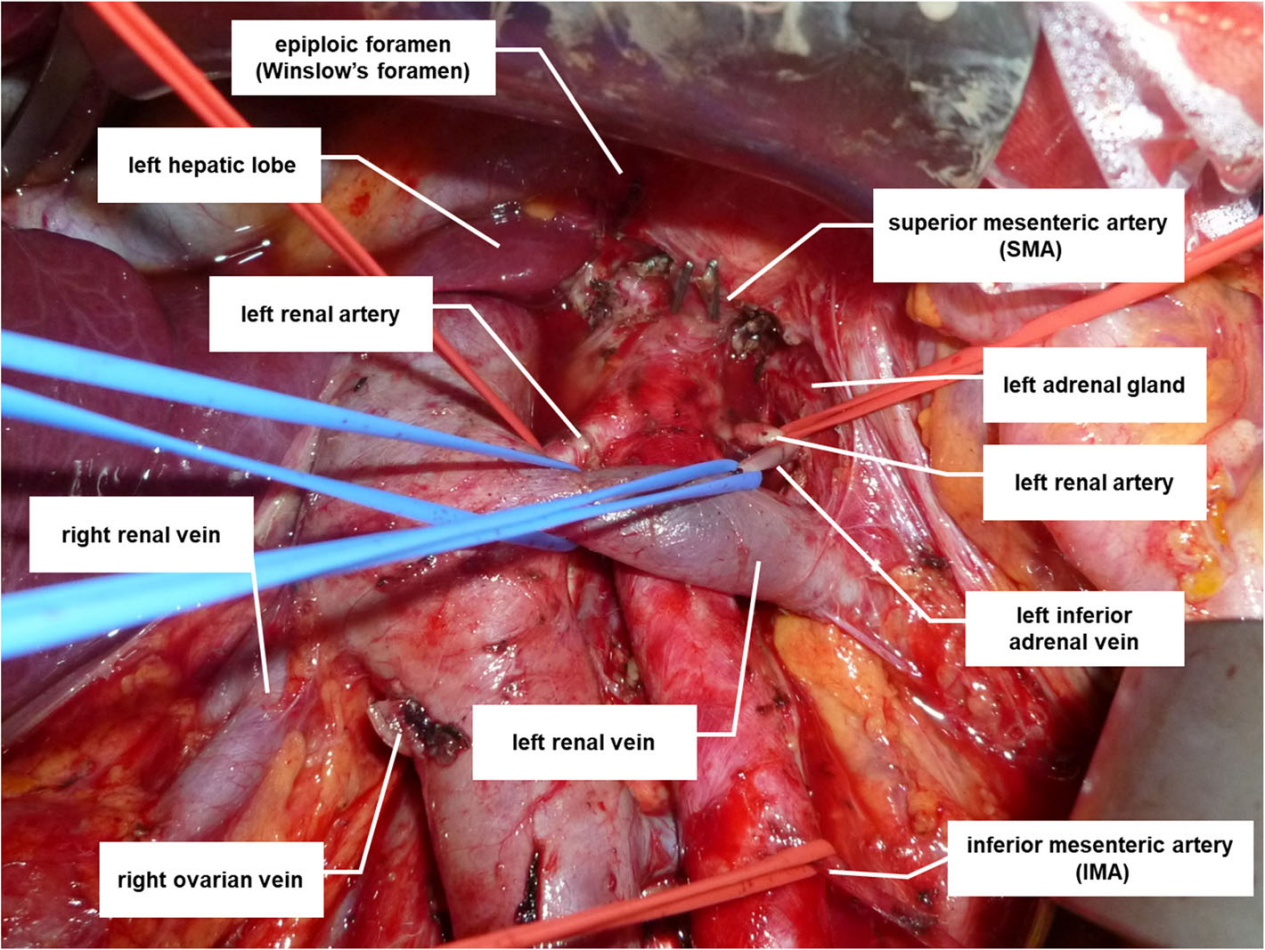
Operating field at the completion of Komiyama’s maneuver for extended para-aortic lymph node dissection

The target region for supra-renal PAN dissection was bounded by the inferior surface of the left lobe of the liver (superiorly), the right border of the IVC (to the right), the medial border of the left adrenal gland (to the left), and the left inferior adrenal vein (inferiorly). Figure 3 shows an intraoperative view after systematic lymph node dissection.

Before the incision was closed, a hyaluronate carboxymethyl cellulose membrane (Seprafilm ®, Sanofi, Paris, France) was placed into the abdominal cavity to prevent postoperative adhesions and drains were inserted into the abdominal cavity. Cefmetazole was administered from the day of surgery until postoperative day 2 to prevent postoperative infection. Low-dose opioid infusion was provided via an epidural catheter until postoperative day 4 for pain management. Subcutaneous administration of enoxaparin sodium (Clexane ®, Sanofi) was started on postoperative day 2 and continued until postoperative day 7 to prevent postoperative deep venous thrombosis or venous thromboembolism.

### Study design

**Fig. 4:**
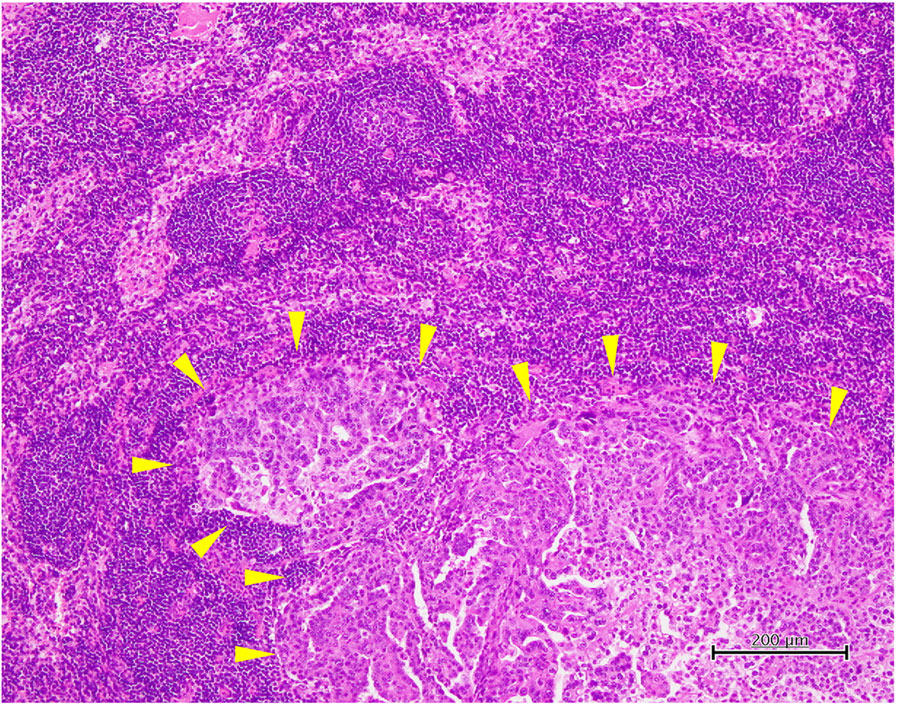
Histopathological findings of metastatic lymph nodes. A supra-renal PAN showed poorly differentiated carcinoma and was diagnosed to be metastasis from high-grade serous ovarian carcinoma (arrows). Hematoxylin and eosin stain

This was a single-center retrospective study. The following patient characteristics and perioperative data were obtained from the medical records: the age, primary tumor site, FIGO stage, tumor histology, serum CA-125 level immediately before surgery, presence or absence of supra-renal PAN measuring ≥ 1 cm on CT before initiation of treatment, presence or absence of ascites at laparotomy, timing of surgery (PDS or IDS), curability of surgery and maximum residual tumor diameter, postoperative Union for International Cancer Control (UICC) pTNM classification (the ypTNM classification was used in patients who received NAC), postoperative duration of hospitalization, operating time, intraoperative blood loss, perioperative complications (from the day of surgery to postoperative day 30), number of dissected lymph nodes, number of metastatic lymph nodes, and locations of metastatic lymph nodes. Formalin-fixed, paraffin-embedded sections of the all dissected lymph nodes were made tissue specimens with the largest section and stained with hematoxylin and eosin. All dissected lymph nodes were examined histologically by pathologists, and metastatic nodes were diagnosed pathologically. When the presence of carcinoma in the parenchyma of the lymph node was observed microscopically, it was diagnosed as lymph node metastasis (Fig. 4). Intraoperative and postoperative complications were evaluated according to the Common Terminology Criteria for Adverse Events (CTCAE) Version 4.03 [4].

This study was conducted in compliance with the Declaration of Helsinki, and the protocol was approved by the ethical committee of Toho University Ohashi Medical Center (approval number H-17044). Informed consent was waived by the board due to the retrospective design of this study.

## Results

From January 2015 to January 2018, 25 patients underwent extended para-aortic lymph node dissection with pelvic lymph node dissection and their characteristics are shown in Table 1. The median number of dissected supra-renal PANs was 5 (range 2–10). In addition, the median number of dissected infra-renal PANs was 21 (range 12–36), and the median number of dissected pelvic lymph nodes (PLNs) was 26 (range 14–55).

**Table 1:** Patient characteristics

		*N* = 25	
		*N*	(%)
Age (years)			
	Median	51	
	Range	31–72	
Preoperative serum CA-125 level (U/ml)*			
	Median	79.6	
	Range	18.9–428.5	
ECOG performance status			
	0	16	64
	1	9	36
FIGO stage			
	I A	4	16
	I C	7	28
	II B	2	8
	III A	1	4
	III C	10	40
	IV A	1	4
Primary tumor site			
	Ovary	21	84
	Peritoneum	3	12
	Fallopian tube	1	4
Histology			
	High-grade serous carcinoma	12	48
	Endometrioid carcinoma	5	20
	Clear cell carcinoma	7	28
	Others**	1	4
Supra-renal PAN enlargement on CT***			
	Yes	2	8
	No	23	92
Ascites at laparotomy			
	Yes	11	44
	No	14	56
Timing of surgery			
	PDS or staging	15	60
	NAC-IDS***	10	40
Operative procedures			
	TH+BSO	25	100
	Omentectomy	25	100
	Pelvic lymphadenectomy	25	100
	Para-aortic lymphadenectomy	25	100
	Pelvic peritoneum stripping	10	40
	Abdominal peritoneum stripping	11	44
	Recto-sigmoidectomy/anastomosis	1	4
	Large bowel resection	1	4
	Diaphragm stripping/resection	8	32
	Splenectomy	1	4
	Liver resection	1	4
	Small bowel resection	1	4
Residual tumors			
	No macroscopic residual tumor	20	80
	Smaller than 1 cm in diameter	5	20
UICC TNM			
	pT1	12	48
	pT2	2	8
	pT3 or ypT3	11	44
No. of dissected lymph nodes			
PLN			
	Median	26	
	Range	14–55	
Infra-renal PAN			
	Median	21	
	Range	12–36	
Supra-renal PAN			
	Median	5	
	Range	2–10	

*Abbreviations*: *CA-125* cancer antigen 125, *NAC* neoadjuvant chemotherapy, *IDS* interval debulking surgery, *ECOG* Eastern Cooperative Oncology Group, *FIGO* International Federation of Gynecology and Obstetrics, *TH* total hysterectomy, *BSO* bilateral salpingo-oophorectomy, *UICC* Union for International Cancer Control, *PLN* pelvic lymph nodes, *PAN* para-aortic lymph nodes

*Including data obtained just before IDS

**Serous carcinoma + endometrioid carcinoma

***Enlargement of supra-renal PAN was defined as at least one node ≥ 1 cm on CT before the start of treatment

***Debulking surgery was performed after 3–4 cycles of chemotherapy (platinum + taxane + bevacizumab)

Supra-renal PAN metastasis was found in 4 of the 25 patients (16.0%). There was no supra-renal PAN metastasis in the 12 patients who had pT1 disease (tumor confined to ovaries or fallopian tube) or the 2 patients with pT2 disease (tumor involves one or both ovaries or fallopian tubes with pelvic extension). In contrast, 4 of the 11 patients (36.4%) with advanced pT3 or ypT3 cancer (tumor involves one or both ovaries or fallopian tubes, or primary peritoneal cancer, with cytologically or histologically confirmed spread to the peritoneum outside the pelvis and/or metastasis to the retroperitoneal lymph nodes) had supra-renal PAN metastasis. All 4 patients underwent NAC and the histological diagnosis was high-grade serous carcinoma in all of them. Only 1 of the 4 patients showed supra-renal PAN enlargement on CT before the start of treatment. There were no patients with isolated supra-renal PAN metastasis (Table 2).

**Table 2:** Details of lymph node metastases in the 25 patients

Primary tumor site	Histological diagnosis	Surgery	Stage (TNM)	Supra-renal PAN		Infra-renal PAN		PLN	
				No. of metastatic lymph nodes	No. of dissected lymph nodes	No. of metastatic lymph nodes	No. of dissected lymph nodes	No. of metastatic lymph nodes	No. of dissected lymph nodes
Ovary	EC	Staging	pT1A N 0 M0	0	8	0	27	0	48
Ovary	EC	Staging	pT1A N0 M0	0	3	0	17	0	24
Ovary	CCC	Staging	pT1A N0 M0	0	4	0	18	0	20
Ovary	CCC	Staging	pT1A N0 M0	0	5	0	16	0	22
Ovary	EC	Staging	pT1C N0 M0	0	4	0	13	0	25
Ovary	EC	Staging	pT1C N0 M0	0	6	0	31	0	32
Ovary	EC	Staging	pT1C N0 M0	0	2	0	21	0	20
Ovary	Others*	Staging	pT1C N0 M0	0	4	0	19	0	21
Ovary	CCC	Staging	pT1C N0 M0	0	5	0	35	0	55
Ovary	CCC	Staging	pT1C N0 M0	0	4	0	32	0	47
Ovary	CCC	Staging	pT1C N0 M0	0	6	0	20	0	33
Ovary	CCC	Staging	pT1A N1 M0	0	4	1	16	1	24
Ovary	CCC	PDS	pT2B N0 M0	0	7	0	28	0	27
Ovary	HGSC	PDS	pT2B N0 M0	0	2	0	17	0	18
Ovary	HGSC	PDS	pT3C N0 M0	0	3	0	26	0	28
Ovary	HGSC	NAC-IDS	ypT3C N0 M0	0	4	0	20	0	22
Ovary	HGSC	NAC-IDS	ypT3C N0 M0	0	3	0	20	0	25
Ovary	HGSC	NAC-IDS	ypT3C N1 M0	2	5	5	19	4	27
Ovary	HGSC	NAC-IDS	ypT3C N1 M0	4	4	16	19	11	23
Ovary	HGSC	NAC-IDS	ypT3C N1 M0	2	4	2	21	3	25
Fallopian tube	HGSC	NAC-IDS	ypT3C N1 M0	0	5	4	26	5	24
Peritoneum	HGSC	NAC-IDS	ypT3C N1 M0	0	5	5	28	6	19
Peritoneum	HGSC	NAC-IDS	ypT3C N1 M0	0	10	11	36	0	30
Peritoneum	HGSC	NAC-IDS	ypT3C N1 M0	4	5	10	15	10	25
Ovary	HGSC	NAC-IDS	ypT3C N1 M1	0	7	0	12	2	14

*Abbreviations*: *EC* endometrioid carcinoma, *CCC* clear cell carcinoma, *HGSC* high-grade serous carcinoma, *Staging* staging surgery, *PAN* para-aortic lymph nodes, *PLN* pelvic lymph nodes, *NAC* neoadjuvant chemotherapy, *IDS* interval debulking surgery

*Serous carcinoma + endometrioid carcinoma

In the 4 patients with supra-renal PAN metastasis, lymph node mapping was performed to evaluate the relation between the locations of the supra-renal and infra-renal PAN metastases and the presence/absence of PLN metastases. It was found that all patients with supra-renal PAN metastasis also had multiple metastases to the infra-renal PANs and PLNs, but there were no correlations between metastases at particular sites (Table 3). In 11 patients with advanced cancer, differences of background factors and perioperative factors were evaluated between the 4 patients who were positive for supra-renal PAN metastasis and the 7 patients who were negative for such metastasis, but no significant differences were found (data not shown).

**Table 3:** Mapping of metastatic nodes in the four patients with supra-renal PAN metastases

			Supra-renal PAN*		Infra-renal PAN*						PLN
					Upper**			Lower***			
Primary tumor site	Histological diagnosis	Stage (TNM)	Metastatic lymph nodes		Metastatic lymph nodes			Metastatic lymph nodes			Metastatic lymph nodes
			G (median)	H (left)	B (right)	G (median)	F (left)	A (right)	C (median)	E (left)	
Ovary	HGSC	ypT3CN1M0	N	P	N	P	P	N	P	N	P
Ovary	HGSC	ypT3CN1M0	P	P	N	P	P	P	P	P	P
Ovary	HGSC	ypT3CN1M0	P	N	N	P	P	N	N	P	P
Peritoneum	HGSC	ypT3CN1M0	P	P	P	P	P	N	P	P	P

*Abbreviations*: *PAN* para-aortic lymph nodes, *PLN* pelvic lymph nodes, *HGSC* high-grade serous carcinoma, *P* positive, *N* negative

*For the locations of A–H, see Fig. 2

**PANs located inferior to the renal veins and superior to the inferior mesenteric artery

***PANs located inferior to the inferior mesenteric artery and superior to the aortic bifurcation

With regard to the feasibility of performing extended para-aortic lymph node dissection, the median operating time was 6.3 h, the median intraoperative blood loss was 675 g, and the blood transfusion rate was 40%. Hypoalbuminemia and anemia were frequent perioperative complications, with the incidence rate of grade 3 hypoalbuminemia being 8% and that of grade 2 hypoalbuminemia being 64%, while the incidence rates of grade 3 and grade 2 anemia were 4% and 44%, respectively. Lymphatic complications also showed a relatively high frequency, and grade 2 lymphatic complications were seen in 20% of the patients. Grade 2 peripheral sensory neuropathy was observed in 12% of the patients, but this was an adverse event due to NAC that persisted after surgery. Similarly, hypertension and proteinuria were noted as adverse events due to the bevacizumab component of NAC that persisted after surgery (Table 4).

**Table 4:** Surgical data and grade 2 or more severe perioperative complications from the day of surgery to postoperative day 30

	*N* = 25		
Duration of hospitalization (days)			
Median	16		
Range	12–22		
Total operating time (minutes)			
Median	380		
Range	295–512		
Intraoperative blood loss (g)			
Median	675		
Range	320–1220		
Complications	Grade 2	Grade 3	≥ Grade 4
	*N* (%)	*N* (%)	*N* (%)
Wound complications (Wound dehiscence)	1 (4)	0	0
Infectious complications*	2 (8)	0	0
Gastrointestinal complications (ileus)	1 (4)	0	0
Urinary complications (incontinence)	1 (4)	0	0
Lymphatic complications**	5 (20)	0	0
Pulmonary complications (pleural effusion)	1 (4)	0	0
Hypoalbuminemia	16 (64)	2 (8)	0
Anemia	11 (44)	1 (4)	0
Hypertension	2 (8)	0	0
Proteinuria	2 (8)	0	0
Peripheral sensory neuropathy	3 (12)	0	0
Other complications***	3 (12)	0	0
Abscess	1 (4)	0	0
Bleeding complications	0	0	0
Thromboembolic complications	0	0	0
Perforation/anastomotic leak	0	0	0
Blood transfusion	10 (40)		

*Urinary tract infection 1, wound infection 1

**Lymphedema 2, lymphocele 1, lymphedema + lymphocele 2

***Chylous ascites 2, alanine aminotransferase (ALT)/aspartate aminotransferase (AST) increased 1

## Discussion

This was the first study to evaluate the actual pattern of metastasis to the para-aortic lymph nodes (PANs) located cephalad to the renal veins in patients with epithelial ovarian cancer, primary peritoneal cancer, or fallopian tube cancer. A total of 25 patients were investigated, including 11 with advanced cancer, so the data are only preliminary because the number of subjects was small. Our findings suggested that supra-renal PANs might be considered as distant lymph nodes rather than regional lymph nodes because none of the patients with early cancer had supra-renal PAN metastasis, while such metastasis was only observed in some of the patients with advanced cancer, all of whom also had widespread multiple metastases to other nodes.

There have already been many reports about infra-renal PAN metastasis in patients with epithelial ovarian cancer. A systematic review of 14 articles showed that 7.1% of patients with early cancer (T1 or T2) had infra-renal PAN metastasis, with 4.3% having both PAN and PLN metastases [5]. When PDS was performed in patients with T3 cancer, the incidence rate of infra-renal PAN metastasis was reported to be 52–84.7% [6–9]. In addition, the incidence rate of infra-renal PAN metastasis was reported to be 47.0–78.5% in patients undergoing IDS, suggesting that PAN metastases were still frequent even after NAC [10–12]. In the present study, infra-renal PAN metastasis was observed in 1 out of 14 patients with T1 and T2 cancer (7.1%) versus 8 out of 11 patients with T3 cancer (72.7%). Although the number of subjects was small, our patients seemed to be a typical population with ovarian cancer. However, we actively performed NAC at our institution, so a high proportion of the patients with advanced disease underwent IDS. Accordingly, we cannot exclude the possibility that the frequency of supra-renal PAN metastasis may have been different if a larger proportion of patients had received PDS. Therefore, it is necessary to increase the number of PDS patients drastically to investigate the frequency of supra-renal PAN metastasis in the future study.

None of our patients had isolated supra-renal PAN metastasis and none of the patients with a clinical diagnosis of early cancer had supra-renal PAN metastasis. In addition, supra-renal PAN metastasis was observed in 36.4% of the patients with a clinical diagnosis of advanced cancer (i.e., the incidence rate was relatively low), and all of the patients with supra-renal PAN metastasis also had widespread multiple metastases of the PLNs and infra-renal PANs. These results suggest that the supra-renal PANs might be considered to be distant lymph nodes rather than regional lymph nodes.

Regarding the feasibility of our method of extended para-aortic lymph node dissection, the operating time was slightly longer, hypoalbuminemia and anemia were frequent, but there were no serious complications, and the procedure was tolerable. In particular, intestinal obstruction and chylous ascites were concerns in relation to this operation, but such events were mild and had a low frequency. In this study, extended PAN dissection was not compared directly with conventional PAN dissection. However, the frequency of complications (including pulmonary complications, infection, intestinal complications, thromboembolism, lymphatic complications, and blood transfusion) was similar to or lower than the rates of perioperative complications reported after conventional PAN dissection [8, 11–13], suggesting that the safety of extended PAN dissection was not inferior to conventional dissection and that dissecting the supra-renal PANs has little negative impact.

Because the current patient population was small, the findings of this study are limited. Accordingly, collection of more data on extended supra-renal PAN dissection in patients with ovarian cancer is required, and further studies should be conducted in a larger population to evaluate the diagnostic significance of supra-renal PAN metastasis. Although there is no doubt about the diagnostic significance of systematic PAN dissection, there have been some negative reports about its therapeutic value [13, 14], suggesting that accumulation of more data is required to allow further discussion of this issue.

## Conclusions

The present findings suggested that supra-renal PAN metastasis should be classified as distant rather than regional metastasis in patients with epithelial ovarian cancer. We also confirmed that our method of extended PAN dissection (Komiyama’s maneuver) was safe and tolerable.

## Data Availability

The data and materials in the current study are available from the corresponding author on reasonable request.
